# Identification of two novel neutralizing nanobodies against swine hepatitis E virus

**DOI:** 10.3389/fmicb.2022.1048180

**Published:** 2022-11-23

**Authors:** Yiyang Chen, Xueting Wang, Meimei Zhang, Jinyao Li, Xueyan Gao, Yuchen Nan, Qin Zhao, En-Min Zhou, Baoyuan Liu

**Affiliations:** Department of Preventive Veterinary Medicine, College of Veterinary Medicine, Northwest A&F University, Xianyang, China

**Keywords:** hepatitis E virus, zoonotic, nanobodies, neutralizing, antiviral

## Abstract

Hepatitis E virus (HEV) is thought to be a zoonotic pathogen that causes serious economic loss and threatens human health. However, there is a lack of efficient antiviral strategies. As a more promising tool for antiviral therapy, nanobodies (also named single-domain antibodies, sdAbs) exhibit higher specificity and affinity than traditional antibodies. In this study, nanobody anti-genotype four HEV open reading frame 2 (ORF2) was screened using phage display technology, and two nanobodies (nb14 and nb53) with high affinity were prokaryotically expressed. They were identified to block HEV ORF2 virus like particle (VLP) sp239 (aa 368–606) absorbing HepG2 cells *in vitro*. With the previously built animal model, the detection indicators of fecal shedding, viremia, seroconversion, alanine aminotransferase (ALT) levels, and liver lesions showed that nb14 could completely protect rabbits from swine HEV infection, and nb53 partially blocked swine HEV infection in rabbits. Collectively, these results revealed that nb14, with its anti-HEV neutralizing activity, may be developed as an antiviral drug for HEV.

## Introduction

Hepatitis E is thought to be an enteric-transmitted zoonotic disease caused by hepatitis E virus (HEV) infection. According to seroprevalence studies from the World Health Organization, there are 20 million HEV infections worldwide, 3.3 million symptomatic cases, and 44,000 deaths (WHO, 2021). The public’s neglection for prevention is responsible for the spread of HEV. Consequently, it is necessary to take urgent, effective public sanitation measures to control HEV infection.

Hepatitis E virus is a positive-sense single-stranded RNA virus that is icosahedral in shape with an approximate diameter of 27–34 nm (Nan et al., 2018). The length of the viral genome is 7.2 kb and contains, in general, three open reading frames (ORFs): ORF1, ORF2, and ORF3 (Kenney and Meng, 2019). ORF1 encodes a 1,700 aa non-structural polyprotein for virus replication, including methyltransferase (MeT), helicase (Hel), and RNA-dependent RNA polymerase (RdRp) (Kenney and Meng, 2019). ORF3 encodes a small phosphorylated protein that is indispensable for HEV replication *in vivo*, and a recent study reported that the ORF3 product had been suggested to be a viroporin that regulates the release of virions (Ding et al., 2017). A novel ORF4 has only been found in the genotype one HEV genome (Nair et al., 2016), which promotes viral replication by inducing endoplasmic reticulum stress. HEV ORF2 (660 aa) encodes the capsid protein and has been used as a key vaccine development target (Chen et al., 2020). The capsid protein is equipped with major immunodominant and neutralizing epitopes (Tang et al., 2011). More than six different neutralizing epitopes on ORF2 have been identified, which mainly occupy the E2 (Protruding) domain (aa 459-602) of the capsid protein (Zhao et al., 2015). In our previous study, three neutralizing monoclonal antibodies recognized linear B-cell epitopes (Chen et al., 2018), and immunization with a polypeptide containing those three epitopes protected rabbits against swine HEV infection (Chen et al., 2020). Thus, as a research target for anti-HEV, the ORF2 protein has broad application prospects.

Nanobodies are at the forefront of antiviral drug research because of their unique advantages (Detalle et al., 2016). Nanobodies, also called single-domain antibodies (sdAbs), consist of one variable domain including 130 amino acids (Muyldermans, 2013). With a small molecule and single-domain nature, nanobodies can recognize special epitopes located in protein crevices (Ma et al., 2021; Zhou et al., 2022). In addition, nanobodies are easy to genetically engineer and mass produce due to their single-domain nature (Vanlandschoot et al., 2011; Hassanzadeh-Ghassabeh et al., 2013). Therefore, nanobodies have the potential to be a novel promising tool for the diagnosis and treatment of infectious diseases (Tarr et al., 2013).

In this study, nanobodies against the genotype four HEV ORF2 protein were screened and prokaryotically expressed. Nb14 and nb53 were shown to block genotype four HEV-absorbing HepG2 cells, and nb14 completely protected rabbits from HEV infection. These results provide a novel drug design and theoretical foundation for HEV antiviral research.

## Materials and methods

### Cells, viruses, sp239 protein, and MAb 1B5

HepG2 cells line were purchased from the American Type Culture Collection (ATCC, Manassas, VA, USA) and cultured in DMEM containing 10% fetal bovine serum and antibiotics (100 U/ml ampicillin and 100 U/ml streptomycin) at 37°C in 5% CO_2_.

The genotype four swine HEV (CHD-SD-sHEV, GenBank No. KF176351) was isolated from a bile sample of a 32-week-old pig, and 10^5^ genomic equivalent (GE)/ml viral stock was obtained from suspensions of fecal and bile samples collected from specific pathogen free (SPF) pigs infected with CHD-SD-sHEV, as previously mentioned (Chen et al., 2018).

The genotype four swine HEV truncated ORF2 protein sp239 (aa 368–606) and the mAb 1B5 (as a positive control) were prokaryotically expressed or generated, as described previously (Chen et al., 2018).

### Selection and identification of sHEV-specific nanobodies

A 4-year-old male Bactrian camel was immunized five times [at 0, 2, 4, 6, and 8 weeks post-inoculation (wpi)] with sp239 protein (2 mg) through subcutaneous injection. The same volume of Freund’s complete (first immunization)/incomplete adjuvant (Sigma-Aldrich, St. Louis, MO, USA) (the next four immunizations) was used. After the last immunization, the anticoagulant blood was collected for nanobody library construction, and biopanning was performed for nanobodies against sp239 selection, as described previously (Sheng et al., 2019). Eventually, all positive clones’ variable domains of camelidae heavy chain-only antibodies (VHH) genes were sequenced, and the nanobodies were classified depending on their CDR3 sequence. Indirect ELISA was performed to detect specific binding between nanobody fragments and sp239, as well as the titration of binding ability.

### Prokaryotic expression of nanobodies against sp239 protein

The selected nanobody genes (nb53 and nb14) were inserted into the pET-21b vector separately with a His-tag. With DNA sequencing confirmation, positive recombinant plasmids were transformed into *Escherichia coli* BL21 (DE3) (TransGen, Beijing, China). After induction of 1.0 mm isopropyl β-D-1-thiogalactopyranoside (IPTG) for 8 h at 37°C, the bacterial cells were collected for ultrasonication. Next, the supernatant and inclusion bodies were collected for target nanobody detection. The soluble nanobodies in the supernatant were purified with a HisTrap Excel column (GE, USA). The inclusion nanobodies were obtained by means of denaturation with 8 M urea, His-tag purification, and renaturation with gradient dialysis (Chen et al., 2018). Analysis of the recombinant nanobodies was performed using sodium dodecyl sulfate-polyacrylamide gel electrophoresis (SDS-PAGE) and Western blotting with horseradish peroxidase (HRP)-labeled anti-His tag antibody (Sino Biological, China).

### Nanobodies blocking sp239 protein attachment to HepG2 cells

The sp239 protein can mimic native HEV particles’ attachment to HepG2 cells (Cai et al., 2016). Therefore, this method was applied to detect the neutralizing abilities of nanobodies. Specifically, 300 μg/ml of nb53 or nb14 was mixed with 50 μg/ml sp239 in phosphate buffer at pH 7.5 and incubated at 37°C for 30 min, and the mixtures were then added to HepG2 cells separately and incubated at 4°C for 30 min. After three washes with phosphate buffered saline (PBS), the cells were lysed with NP40 lysis buffer (Boster Biological Technology, China) supplemented with protease and phosphatase inhibitors. Finally, the amount of sp239 attached to HepG2 cells was detected by Western blotting (Chen et al., 2018).

### Immunofluorescence focus assay and flow cytometry neutralization assay

The immunofluorescence focus assay (IFA) and flow cytometry neutralization (FCM) assay were used to analyze nanobodies blocking sp239 attachment to HepG2 cells. HepG2 cells were cultured on coverslips and incubated with sp239 or a mixture of sp239 (10 μg/ml) and either 300 μg/ml of nb14, nb53, 1B5, or nb-anti-porcine circovirus type 2 (PCV2) for 30 min at 4°C. After washing three times with PBS, the cells were fixed with 4% paraformaldehyde (Sigma-Aldrich, St. Louis, MO, USA) and permeabilized with 0.3% Triton X-100 (Sigma-Aldrich, St. Louis, MO, USA) in PBS. Fixed cells were blocked with 10% goat serum in PBS for 1 h at 25°C, incubated with primary anti-sp239 mAb 3E8 (1:2,000) for 1 h at 25°C, and labeled with Alexa Fluor 555 goat anti-mouse IgG (1:5,000; Thermo Fisher Scientific, Waltham, MA, USA) for 1 h at 25°C. After three washes, the cells were stained with DAPI (Invitrogen, Carlsbad, CA, USA), and the fluorescence signals were detected with a Leica microscope (LSM 780; Carl Zeiss, Oberkochen, Germany).

For FCM, cells were collected and subjected to FCM (BD FACSAria™ III 03141313, Biosciences, San Jose, CA, USA) using a HyperCyt loader (UNC, USA), and the data were analyzed with FlowJo.V10 software (BD Biosciences, San Jose, CA, USA). The primary and secondary antibodies used in FCM were the same as those used for IFA.

### Nanobodies blocking natural swine hepatitis E virus infection in HepG2 cells by real-time RT-polymerase chain reaction

Hepatitis E virus can be grown in HepG2, A549, and PLC/PRF/5 cells at low levels (Fu et al., 2019), but a method for HEV quantitation conducted with real-time reverse transcription-polymerase chain reaction (RT-PCR) was developed by Tang et al. (2015). Neutralization tests were performed to identify whether the nanobodies can block genotype four swine HEV replication in HepG2 cells. Briefly, nb14, nb53, 1B5, or nb-anti-PCV2 at different amounts (1, 0.1, and 0.01 mg) was mixed with 10^5^ copies of swine HEV and incubated at 37°C for 30 min, while the virus was only used as the positive control. Each mixture was then separately added to HepG2 cells (10^6^ cells per well) and incubated at 37°C for 30 min. After three washes with PBS, the RNA from total HepG2 cells was extracted with TRIzol reagent (TransGen, China) by following the instructions. In the last step, TaqMan RT-PCR was conducted to quantify HEV RNA with the QuantiTect Probe RT-PCR kit master mix (Qiagen, Valencia, CA, USA).

### Neutralization assays in rabbits

In a previous study, an animal model for swine HEV infecting SPF rabbits was developed (Chen et al., 2018, 2020). Therefore, a total of 30 8-week-old SPF *New Zealand White* rabbits (Dossy Laboratory Animal Technology Co., Ltd., Chengdu, China), with initial body weights averaging 1.5 kg (SD, ± 1.67 kg), were evenly divided into six groups. Each rabbit was kept in a separate cage under constant temperature and humidity. Before immunization, each rabbit was confirmed to be serum negative for anti-HEV antibodies and fecal negative for HEV RNA after indirect ELISA and nested RT-PCR detection, respectively.

The swine HEV stock (2 × 10^4^ GE) prepared from a 10% fecal stock was incubated with nb14, nb53, 1B5, or nb-anti-PCV2 (2 mg, 2 mg/ml), including the virus-only and PBS groups. The mixtures were incubated for 1 h at 25°C and then overnight at 4°C. After a week, five rabbits in each group were injected with the mixtures *via* the ear vein. The serum and fecal samples were collected weekly after inoculation until 12 weeks for antibody levels, viremia, or fecal virus shedding tests. Moreover, the serum samples were also used for levels of ALT detection.

### Qualitative detection of swine hepatitis E virus RNA using RT-polymerase chain reaction

Swine HEV RNA in fecal and serum samples was detected using RT-nPCR as described previously (Huang et al., 2002). Specifically, viral RNA was extracted from 200 μl sera or 10% fecal volume using TransZol Reagent (TransGen, China) according to the manufacturer’s instructions. Reverse transcription and the first PCR were performed for RT-nPCR with the PrimeScript™ One Step RT-PCR Kit (Takara, Tokyo, Japan), and the second PCR was performed with TransTaq High Fidelity DNA polymerase (TransGen, Beijing, China). Finally, the PCR products were analyzed with gel electrophoresis.

### Detection of anti-swine hepatitis E virus antibodies and alanine aminotransferase levels

The anti-swine HEV antibodies in serum samples were detected by indirect ELISA with sORF2-C as a coating antigen (Chen et al., 2016). Briefly, the purified swine HEV ORF2 protein sORF2-C (aa 393–660) was coated on 96-well plates at 4°C overnight. After blocking and washing, the collected serum samples (100 μl/well) were added to each well and incubated for 1 h at 25°C. After three washes, HRP-conjugated goat anti-rabbit IgG (Jackson ImmunoResearch, West Grove, PA, USA) (diluted 1:4,000, 100 μl/well) was added to the wells and incubated for 1 h at 25°C. After the next three washes, 100 μl of 3,3’,5,5’-tetramethylbenzidine (TMB) was added and incubated for 15 min at 25°C. As the final step, the colorimetric reaction was terminated by 3 M H_2_SO_4_ (50 μl/well), and the OD_450nm_ value was read by an automatic ELISA microplate reader.

The ALT levels in plasma samples reflected the situation of liver damage and were measured following the standard methods of the manufacturer’s instructions (Hitachi 912; Roche, USA). Rabbits were considered positive for hepatitis if post-challenge ALT levels exceeded pre-challenge ALT levels by more than twofold (Ma et al., 2010).

### Microscopic and transmission electron microscopy hepatic lesions

During necropsy, liver tissues were collected and fixed in 10% neutral-buffered formalin and then prepared for histological examination. Histopathological lesions in the liver were evaluated according to previous experiments (Chen et al., 2020). Furthermore, the hepatic lesions were observed for ultrastructure by transmission electron microscopy (TEM), and the detailed operation was performed as described previously (Liu et al., 2020).

## Ethics statement

All animal experimental procedures were executed according to the guidelines of the Northwest A&F University Institutional Committee for the Care and Use of Laboratory Animals, and the study was approved by the Committee on Ethical Use of Animals of Northwest A&F University (AE324812).

## Results

### Screening and sequencing of nanobodies against sp239 protein

**FIGURE 1:**
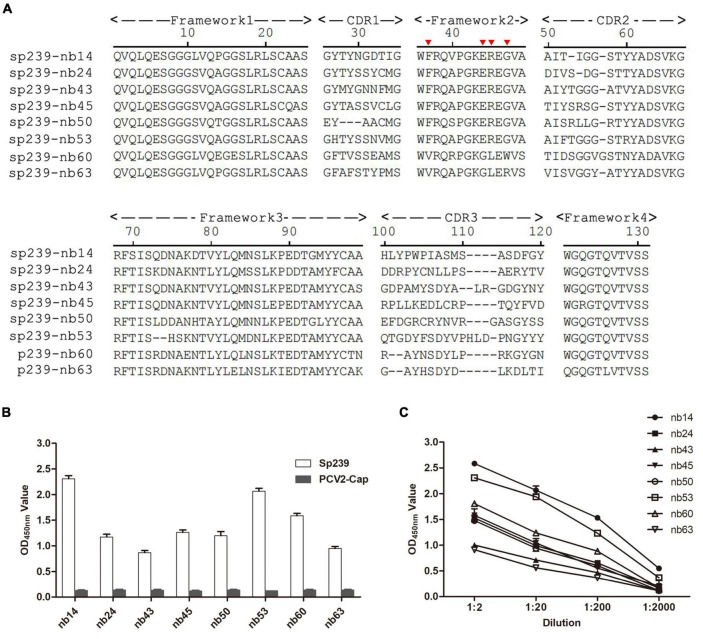
Screening of nanobodies against the hepatitis E virus (HEV) sp239 protein. **(A)** Alignment of the amino acid sequences of eight isolated nanobodies. The numbering of frameworks and complementarity-determining regions (CDRs) is based on previous methods (Kabat et al., 1991). **(B)** The specific binding between sp239 and nanobodies with indirect ELISA. The PCV2-cap protein was used as a control. **(C)** Titration of the nanobodies. Data were processed with triplicate experiments and are presented as the means ± SDs.

A phage display VHH library was constructed from an sp239 protein-immunized camel, and 89/96 antibody fragments were specifically combined with sp239. Under sequence alignment and consolidation, a total of eight unique nanobodies (nb14, nb24, nb43, nb45, nb50, nb53, nb60, and nb63) were screened and identified according to the CDR3 region. Except for nb60 and nb63, other nanobodies had conserved residues Val37, Gly44, Leu45, and Trp47, which were considered hydrophilic amino acids (Figure 1A, red triangle). With the PCV2-cap protein as a negative control, all nanobodies only reacted with sp239 by iELISA (Figure 1B). In addition, the iELISA results indicated that nb14 and nb53 exhibited the highest binding activity (Figure 1C). Consequently, nb14 and nb53 were selected for further research.

### Expression and purification of nanobodies

**FIGURE 2:**
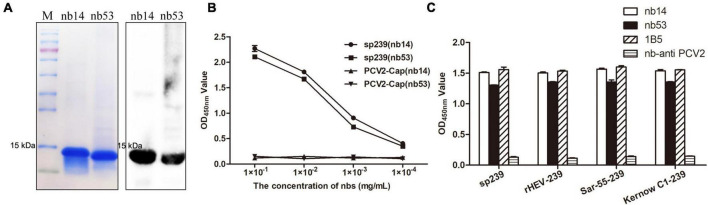
Analysis of purified nanobodies. **(A)** His-tagged nb14 and nb53 with a predicted size of 15 kDa were detected by SDS-PAGE and Western blotting. **(B)** Detection of the binding activities of nb14 or nb53 to sp239 by iELISA. The PCV2-cap protein was used as a negative control. Data were processed with triplicate experiments and are presented as the means ± SDs. **(C)** The nb14 and nb53 interact with sp239, rHEV-239, Sar-55-239, and Kernow C1-239 protein by iELISA. The 1B5 and nb anti-PCV2 antibodies were used as positive and negative controls, respectively.

Nb14 and nb53 with His-tags were expressed in an *E. coli* prokaryotic system as soluble proteins and inclusion bodies, respectively. After affinity purification with a Ni column (additional steps of denaturing in urea, gradient dialysis for nb53 as inclusion body), the SDS-PAGE and Western blotting results showed that nb14 and nb53 appeared with a predicted size of approximately 15 kDa (Figure 2A). As a coating antigen, the sp239 protein was used to detect the binding ability with nb14 and nb53, whereas PCV2-cap was used as a negative control. The iELISA revealed that these two recombinant nanobodies specifically reacted with sp239 and shared good binding abilities (Figure 2B). Meanwhile, the results also showed that nb14 and nb53 reacted with capsid proteins (aa 368–606) of the genotype three rabbit HEV strain (CHN-SX-rHEV) and the genotype one and three human HEV strains (Sar-55 and Kernow C1) (Figure 2C).

### Nanobodies blocked sp239 attachment and natural hepatitis E virus infection of HepG2 cells

**FIGURE 3:**
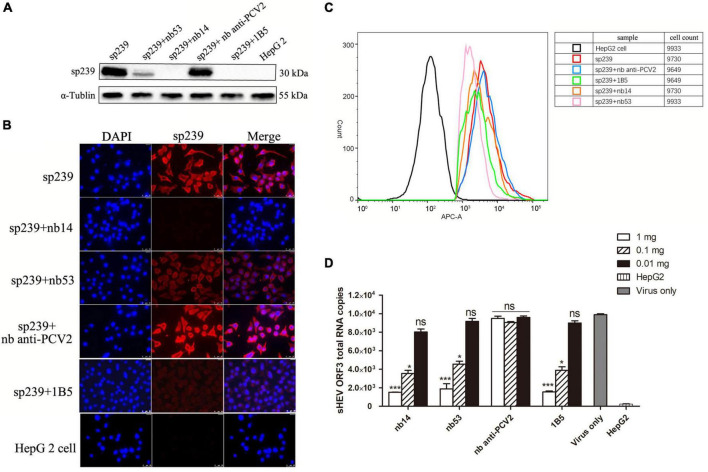
Nanobodies blocking sp239 absorption or natural swine HEV particle infection of HepG2 cells. Nanobodies blocking sp239 attachment to HepG2 cells followed by detection with Western blotting **(A)**, immunofluorescence focus assay (IFA) **(B)**, and flow cytometry neutralization (FCM) **(C)** assays. **(D)** Nanobodies blocking swine HEV infection of HepG2 cells by real-time RT-PCR. The nb anti-PCV2 and the MAb 1B5 served as negative and positive controls, respectively. Data are the mean ± SD of results based on triplicate experiments. *P*-values were calculated with student’s *t*-test. *P < 0.05; **P < 0.01; ***P < 0.001; ns, not significant.

As shown in the first lane of the Western blotting results (Figure 3A), the top row of IFA (Figure 3B) and FCM data (Figure 3C, red line), the sp239 protein could adsorb HepG2 cells well; hence, it was used to detect the neutralizing abilities of nanobodies *in vitro* instead of HEV particles. 1B5 and another nanobody against PCV2 served as positive and negative controls, respectively. Western blotting results also showed that the amounts of sp239 attached to HepG2 cells were reduced after incubation with nb53, nb14, and 1B5 compared with the nb anti-PCV2 or only sp239 (Figure 3A). Consistently, according to the IFA results, the fluorescence intensity of sp239 absorbed by HepG2 cells after incubation with nb53, nb14, and 1B5 was significantly weaker than that after incubation with only sp239 or sp239 incubated with nb anti-PCV2 (Figure 3B). The FCM results also revealed that sp239 premixed with nb53, nb14, or 1B5 (pink, orange, and green curve) exhibited slight binding compared to sp239 pre-incubated with nb anti-PCV2 (blue curve) (Figure 3C). These results suggested that nanobodies could block the adsorption of sp239 protein to HepG2 cells.

Compared with the virus-only group at 1 and 0.1 mg, HEV RNA copy numbers were significantly decreased in the nb14, nb53, and 1B5 groups, and the nb anti-PCV2 groups showed the same copy numbers as the virus-only group (Figure 3D). These results indicate that nb14 and nb53 protect HepG2 cells from HEV infection in a dose-dependent manner.

### Nanobodies neutralize swine hepatitis E virus infection in rabbits

**FIGURE 4:**
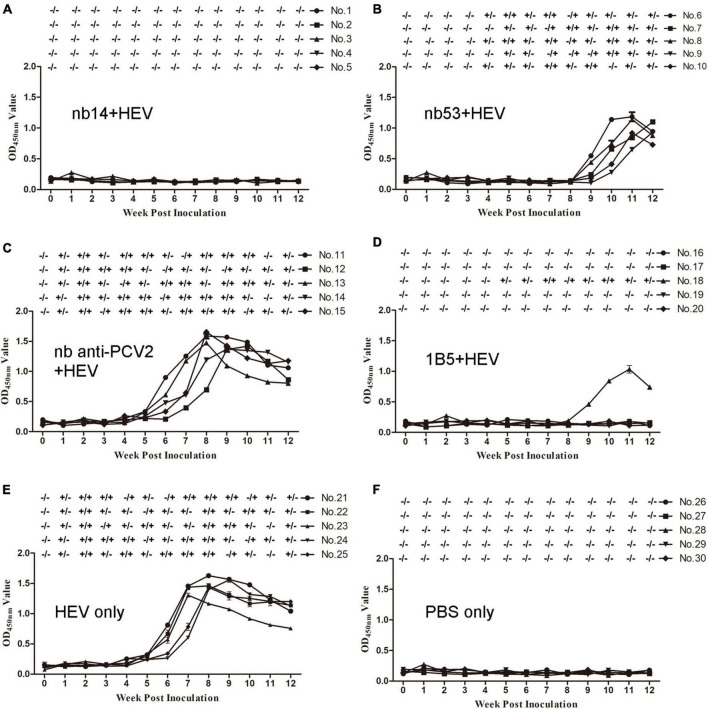
Analysis of viremia, fecal shedding, and seroconversion in SPF rabbits inoculated with different virus mixtures. **(A–D)** Rabbits inoculated with swine HEV mixed with nb14, nb53, nb anti-PCV2, and 1B5. **(E,F)** Rabbits inoculated with only CHN-SD-sHEV or PBS. Serum and fecal samples were collected before inoculation and weekly. ±, positive or negative HEV RNA in fecal or serum samples.

Before inoculation, all rabbits used in the experiment were negative for HEV RNA and anti-HEV IgG antibodies. All rabbits in the virus-only (Figure 4E) group and nb anti-PCV2 (Figure 4C) group seroconverted at 6 wpi. Conversely, in the groups inoculated with nb14 (Figure 4A), 1B5 (Figure 4D), and PBS (Figure 4F), all rabbits (except for No. 18 in the 1B5 group) were seronegative for anti-HEV antibody throughout the study. The seroconversion of rabbits inoculated with nb53 and No. 18 began at 9 wpi and was delayed by 3 weeks compared with the virus-only group (Figures 4B,D).

Fecal shedding and viremia were detected at 1 or 2 wpi and lasted until 12 or 10 wpi (Figure 4E) in the virus-only group and the nb anti-PCV2 group as a negative control (Figure 4C). No fecal shedding or viremia was detected in rabbits of the nb14 (Figure 4A), 1B5 (except for No. 18) (Figure 4D), and PBS (Figure 4F) groups. The fecal shedding and viremia of rabbits in the nb53 group and No. 18 began at 4 and 5 wpi (Figures 4B,D), and there was a delay compared to the virus-only group. These results indicated that nb14 entirely neutralized swine HEV infection in rabbits, while nb53 can also neutralize HEV *in vivo*, but not as obviously as nb14.

### Evaluation of alanine aminotransferase levels in serum samples

**FIGURE 5:**
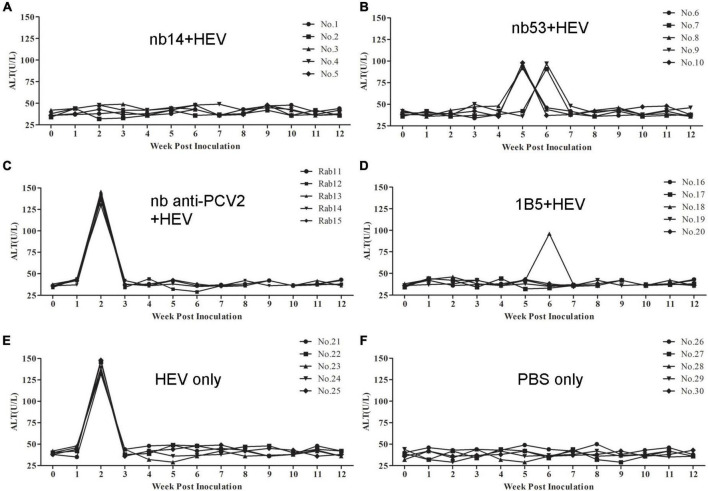
The alanine aminotransferase (ALT) levels of liver enzymes in the serum of different groups of rabbits. **(A–D)** Rabbits inoculated with swine HEV mixed with nb14, nb53, nb anti-PCV2, or MAb 1B5. **(E,F)** Rabbits inoculated with only swine HEV or PBS.

The ALT level of each rabbit was detected weekly. In the nb14, 1B5 (except for No. 18), and PBS groups, no obvious ALT level changes appeared in rabbits (Figures 5A,D,F). Conversely, a peak ALT level of 136-146 U/L or 132-148 U/L occurred at 2 wpi in all rabbits of the nb anti-PCV2 or virus-only group (Figures 5C,E). In rabbits of the nb53 group and No. 18 (Figures 5B,D), the ALT level of 91–98 U/L peaked at 5 or 6 wpi. Compared with the virus-only group, various degrees of descent and delays in the ALT level peak appeared in the nb53 group.

### Microscopic and transmission electron microscopy hepatic lesions

**FIGURE 6:**
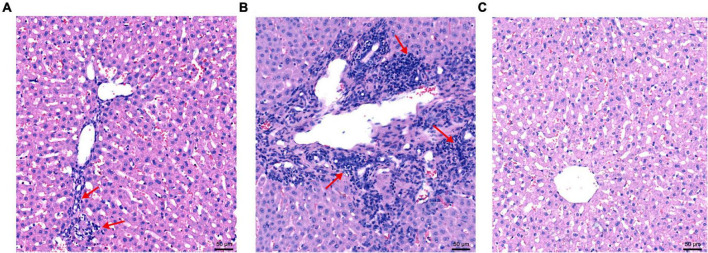
The microscopic lesions of liver sections in necropsied rabbits. **(A)** Liver tissues from rabbits with slight lymphocytic venous periphlebitis in the nb53 group. **(B)** Severe lymphocytic venous periphlebitis in hepatocytes in virus only or nb anti-PCV2 groups. **(C)** No obvious pathological signs of microscopic lesions in the rabbits of the nb14 and 1B5 groups.

**FIGURE 7:**
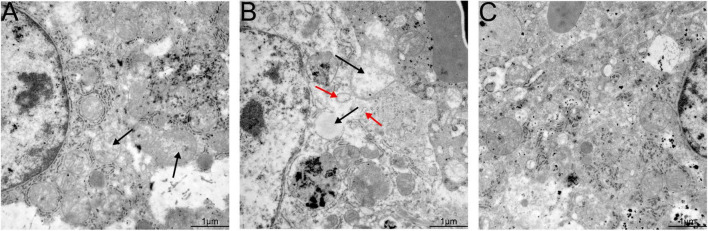
Transmission electron microscopy (TEM) of liver tissues from necropsied rabbits. **(A)** Slight ultrastructural pathological changes, such as the loss of mitochondrial cristae, in the rabbits of the nb53 group. **(B)** Serious mitochondrial cristae loss and swollen mitochondria in hepatocytes in rabbits of the virus only or nb anti-PCV2 groups. **(C)** No visible ultrastructural changes in the rabbits of the nb14 and 1B5 groups.

Hepatic lesions in liver tissue and ultrastructural pathological changes in hepatocytes were observed as indicators of hepatitis with histological examination and TEM. Specifically, slight lymphocytic venous periphlebitis (Figure 6A, red arrow) was observed in liver sections of rabbits in the nb53 group and No. 18, and slight ultrastructural changes, such as the loss of mitochondrial cristae, also emerged in these rabbits (Figure 7A, black arrow). Focally intense lymphocytic venous periphlebitis (Figure 6B, black arrow) occurred on liver sections of rabbits in the virus-only and nb anti-PCV2 groups, and the severe loss of mitochondrial cristae, swollen mitochondria (Figure 7B, black arrow), and endoplasmic reticulum (Figure 7B, red arrow) were also observed. All rabbits in the nb14, 1B5 (except for No. 18), and PBS groups had no evident liver microscopic lesions or ultrastructural changes (Figures 6C,7C). These results suggest that nb14 could protect liver tissue and hepatocytes from injury caused by HEV infection and that nb53 has partial protection.

## Discussion

The HEV causes hepatitis E through intestinal transmission and is considered zoonotic, and pigs serve as the main natural reservoir (Doceul et al., 2016). Genotype four HEV (HEV-4) is widespread in healthy pig herds around the world (Sooryanarain and Meng, 2020). HEV does not cause obvious symptoms in pigs, but severe liver hemorrhage and necrosis occur with massive lymphocytic infiltration in various organs, including the liver, lung, and intestine, when HEV is coinfected with porcine reproductive and respiratory syndrome virus (PRRSV) or PCV2 (Mao et al., 2013; Jackel et al., 2019). There are unpredictable risks to the healthy development of the pig industry. In addition, food-borne HEV infection has become an important factor in HEV infection in the population and poses a threat to human health (Sridhar et al., 2017). Therefore, it is particularly important to block the spread of swine HEV in pig herds.

The HEV ORF2 encodes virus capsid protein and contains the main immunodominant epitopes of virion (Yin et al., 2018). There was a single serotype on HEV albeit four major genotypes, with >90% amino acid sequence identity among them (Kamar et al., 2012). In this research, the iELISA results showed that nb14 and nb53 bound to the ORF2 protein of swine HEV-4, human HEV-1 (Sar-55), human HEV-3 (Kernow C1), or rabbit HEV-3 (Figure 2C). These results may reveal that the same neutralizing ability of nanobodies may apply to other HEV strains.

In general, mammalian cells or insect cell secreted the protein and ensured that the folding of proteins was correct. However, the problems of expensive medium, slow growth, impurities from the serum used in the growth medium, difficult purification, and expensive final product blocked the further application (Khodarovich et al., 2013). In our study, the prokaryotic expression system was used to express nanobodies for the advantages of easily and inexpensively grown cells, inexpensive growth media, easy purification, and rapid achievement of high levels of expression, and the gradient dialysis and renaturation were performed for protein refolding correctly. A nanobody against PRRSV with a prokaryotic expression system displayed antiviral neutralizing activity that had been reported by Wang et al. (2019).

As potential drugs for antiviruses, nanobodies have the advantages of simple structure, good solubility and stability, high specificity, and convenient gene modification (Muyldermans, 2021). Due to their small size, most nanobodies recognize conformational epitopes located in protein crevices (Tarr et al., 2013). In this study, nb14 and nb53 had good capability for binding to sp239 according to the ELISA results (Figure 2B), and Western blotting showed that there was no binding between sp239 and nb14 or nb53 (data not shown), which may be because the sp239 protein was denatured and became a linear structure. In fact, a series of truncated sp239 proteins were designed and prokaryotically expressed for the recognized epitopes nb14 and nb53, but the truth is that nb14 or nb53 did not bind to any truncated protein (data not shown). These results suggest that nb14 and nb53 may recognize the conformational epitopes on sp239. Furthermore, more structural biological studies should be performed.

In our previous study, three monoclonal antibodies recognizing linear B-cell epitopes were identified as neutralizing swine HEV in rabbits, but one or two rabbits in the groups inoculated with neutralizing MAbs showed low-activity infection (Chen et al., 2018). Similar results were observed for No. 18 in the 1B5 group in this study. Conversely, no fecal shedding, viremia, antibody, or ALT level changes occurred in any of the rabbits inoculated with nb14. The possible reasons for this difference are the inefficient neutralization by an MAb and the neutralizing ability of conformational epitopes being superior to linear epitopes.

It is well known that the mitochondria and endoplasmic reticulum play an important role in cellular reproduction. HEV replication is mediated by endoplasmic reticulum stress and is sustained by the mitochondrial electron transport chain (Nair et al., 2016; Qu et al., 2019). In our previous findings, the loss of mitochondrial cristae, swollen mitochondria, and endoplasmic reticulum were observed in the hepatocyte cells of silkie (*Gallus gallus*, a kind of special economic chicken) infected with HEV by TEM (Liu et al., 2020). In this study, the same ultrastructural pathological changes in hepatocyte cells were also found in rabbits infected with swine HEV (Figure 7C). These results may suggest that the mitochondria and endoplasmic reticulum are important for HEV replication.

In summary, two nanobodies (nb14 and nb53) were generated from a camel VHH library, and they exhibited neutralizing activities in HepG2 cells infected with sp239 protein or swine HEV. Furthermore, nb14 completely protected rabbits from HEV infection, and nb53 exhibited partial protection. Thus, this study suggests that nb14 has the potential to help design antiviral drugs for zoonotic HEV.

## Data availability statement

The raw data supporting the conclusions of this article will be made available by the authors, without undue reservation.

## Ethics statement

The animal study was reviewed and approved by the Northwest A&F University Institutional Committee for the Care and Use of Laboratory Animals.

## Author contributions

YC and BL designed the experiments and drew the scheme and figures. YC and XW performed the experiments. MZ, JL, XG, YN, and QZ contributed to the reagents, materials, and analysis tools. YC, BL, and E-MZ analyzed the data and wrote the manuscript. All authors read and approved the final manuscript.
